# A new perspective on mesenchymal stem cells and their derivatives in alleviating cerebral ischemia-reperfusion injury dynamically regulating mitochondrial function to remodel the immune network

**DOI:** 10.1038/s41419-026-08834-7

**Published:** 2026-05-08

**Authors:** Lizheng Huang, Yuqian Wang, Chunyi Li, Xiaojing Wang, Anqi Xiao, Fanglei Han, Jinlan Jiang

**Affiliations:** 1 Department of Anesthesiology, China-Japan Union Hospital, Jilin University, Changchun, Jilin China; 2 Scientific Research Center, China-Japan Union Hospital, Jilin University, Changchun, Jilin China; 3 Institute of Antler Science and Product Technology, Changchun Sci-Tech University, Changchun, Jilin China; 4 Changchun University of Science and Technology, Changchun, Jilin China; 5 Ankangyuan Biotech (Jilin) Co. Ltd, Changchun, Jilin China

**Keywords:** Mesenchymal stem cells, Cell death and immune response

## Abstract

Cerebral Ischemia-Reperfusion Injury (CIRI) is a common pathological process in ischemic stroke. Its core detriment lies in the cascade of subsequent injuries triggered by blood flow restoration after cerebrovascular recanalization, primarily including oxidative stress burst, calcium overload, immune-inflammatory imbalance, and mitochondrial dysfunction, ultimately leading to massive neuronal apoptosis and necrosis. Mitochondria, as central hubs of cellular energy metabolism and apoptosis regulation, exhibit functional disturbances that represent the initiating and core link in CIRI. CIRI triggers complex remodeling of the immune network. This dysregulated immune-inflammatory response forms a vicious cycle with mitochondrial dysfunction, exacerbating brain tissue damage. Mesenchymal Stem Cells (MSCs) and their derivatives have demonstrated significant potential in treating CIRI due to their potent paracrine and immunomodulatory functions. MSCs can repair neuronal functional basis by enhancing mitochondrial biogenesis, improving energy metabolism, inhibiting mitochondrial pathway-mediated apoptosis, restoring dynamic balance, and promoting normal mitophagy. Although existing reviews have explored the role of MSCs or mitochondrial function in CIRI, few studies have systematically integrated the bidirectional regulatory relationship between dynamic mitochondrial function and immune network remodeling. Furthermore, an in-depth analysis of the temporal therapeutic effects of MSCs and their derivatives across different pathological stages of CIRI is lacking. To address this gap, this review proposes a therapeutic strategy: MSCs exert synergistic neuroprotective effects by improving mitochondrial function through multiple targets while systematically reshaping the imbalanced immune network. This review summarizes the latest evidence regarding the temporal therapeutic role of MSCs via the “immune-mitochondrial” axis at different stages of CIRI. It elucidates the pivotal role of mitochondria as a central hub connecting metabolic crisis and immune storm, and untangles novel mechanisms such as MSC-mediated mitochondrial transfer and immune cell metabolic reproprogramming, providing a theoretical foundation for developing novel stroke therapies based on cell therapy.

## Facts

- Mitochondrial function serves as a central hub linking metabolic crisis and immune storm, rather than merely acting as an energy factory.
- MSCs can perform intercellular energy rescue via “mitochondrial transfer”, representing a novel mechanism of “intercellular metabolic support.”
- The “immune–mitochondrial axis” constitutes a new therapeutic target for CIRI, and MSCs achieve “temporal therapy” through dynamic regulation of this axis.
- MSCs modulate immune cell polarization by metabolic reprogramming-such as promoting OXPHOS and suppressing glycolysis-thereby influencing inflammatory outcomes.
- MSC-derived products can serve as a “cell-free therapy” alternative to MSCs, offering improved safety profiles and regulatory potential.

## Open questions

- What is the “optimal therapeutic time window” for MSCs and their derivatives across different pathological stages of CIRI, and how can precise temporal intervention be achieved?
- How do mitochondria derived from MSCs integrate, survive, and function within recipient cells? Is there a risk of immune rejection or functional alteration?
- How can the standardized and large-scale production of MSC-derived products (e.g., exosomes) be achieved, and how can their brain-targeted delivery efficiency be improved?
- Are there druggable key molecular nodes within the “immune–mitochondrial axis” that could be targeted for the development of non-cell-based therapies?

## Introduction

Currently, stroke is the leading cause of acquired disability among adults worldwide and the primary cause of disability and mortality among Chinese residents [1]. Stroke is an acute cerebrovascular disease characterized by sudden rupture and bleeding of cerebral vessels or obstruction of cerebral blood flow leading to ischemia in corresponding brain regions, resulting in brain tissue damage. The occurrence rate of ischemic stroke is significantly higher than that of hemorrhagic stroke, accounting for 67.3%–80.5% of all stroke cases [2]. A major goal in the treatment of ischemic stroke is the restoration of cerebral blood flow. However, the reperfusion of blood flow after ischemia can exacerbate brain tissue damage, a phenomenon known as cerebral ischemia-reperfusion injury (CIRI).

CIRI refers to the pathological phenomenon in which cellular damage and death are aggravated after the restoration of blood reperfusion to the brain. Its underlying mechanisms evolve dynamically as the disease progresses [3]. During the ischemic phase, inadequate oxygen supply leads to gradual depletion of cellular energy reserves, while compensatory metabolic pathways are activated, resulting in the accumulation of acidic metabolites within cells and subsequent cellular injury [4]. Upon the initiation of reperfusion, a cascade of injuries-including oxidative stress, calcium overload, inflammatory responses, and apoptosis-is triggered [5, 6]. This time-dependent pathological progression necessitates the consideration of dynamically evolving intervention strategies targeting CIRI.

The core pathological basis of CIRI is closely associated with mitochondrial dysfunction and immune-inflammatory responses. The vicious cycle formed by these two components, termed the “immune-mitochondrial” axis, exhibits distinct dominant characteristics at different stages of the disease course [7–9]. As the central organelle for intracellular energy metabolism, mitochondria exhibit functional abnormalities during the acute phase of ischemia under ischemic and hypoxic conditions. The interruption of oxygen and glucose supply halts the oxidative phosphorylation (OXPHOS) process, leading to a rapid decline in ATP synthesis and triggering an energy crisis [10, 11]. Concurrently, electron leakage from the electron transport chain results in massive generation of reactive oxygen species (ROS), which exacerbates oxidative damage and induces apoptosis and necrosis [12–14]. Upon entering the reperfusion phase, mitochondrial function fails to recover promptly. The impaired electron transport chain induces more severe ROS outbursts and loss of membrane potential, triggering the opening of the mitochondrial permeability transition pore (mPTP) and the release of pro-apoptotic factors such as cytochrome C, thereby initiating apoptosis [15, 16]. This process becomes a critical factor driving cell death. Additionally, the release of mitochondrial DNA activates innate immune pathways, further aggravating the inflammatory response. At the immune level, damage-associated molecular patterns (DAMPs) released by damaged neurons during the acute ischemic phase activate microglia and attract peripheral immune cells, including neutrophils and monocytes/macrophages, to infiltrate the brain parenchyma through the compromised blood-brain barrier (BBB) [17–19]. These immune cells release large quantities of pro-inflammatory cytokines, triggering a “cytokine storm” that disrupts the blood-brain barrier, exacerbates cerebral edema, and expands the scope of brain injury. Ultimately, this leads to the extension of the infarct area into the surrounding penumbra [20–22]. Furthermore, the immune response transitions from innate immune activation in the acute phase to deep involvement of adaptive immunity in the subacute phase, while immunosuppression and impaired tissue healing are often observed during the recovery phase.

Currently, clinical treatment options for CIRI remain relatively limited, posing numerous challenges in clinical management. Vascular recanalization, such as intravenous thrombolysis or mechanical thrombectomy within the therapeutic time window, serves as the primary approach for restoring blood flow [23]. Although these methods can reestablish perfusion, they carry risks of hemorrhagic transformation and reperfusion injury. Moreover, many patients cannot benefit from them due to delayed hospital admission. Conventional neuroprotective agents-including edaravone [24, 25], anti-inflammatory statins [26], and Ginkgo biloba extracts [27]-as well as traditional Chinese medicine therapies, aim to mitigate subsequent injury but exhibit notable limitations. These agents often target single pathways and are inadequate for addressing the complex, multi-pathway, multi-target mechanisms involved in CIRI. In addition, the blood–brain barrier restricts drug delivery efficiency, thereby limiting both clinical efficacy and safety. Consequently, developing novel therapies capable of synergistically modulating multiple pathological processes and adapting to different stages of the disease has become a major research focus in this field.

In recent years, numerous reviews have explored the pathological mechanisms of CIRI and the therapeutic potential of MSCs. However, existing research still exhibits significant limitations and gaps, which constitute the core innovative entry point of this review. Some reviews only separately discuss the roles of mitochondrial dysfunction or immune-inflammatory responses in CIRI, neglecting the core relationship of their interaction and formation of a vicious cycle. They fail to systematically analyze the “immune-mitochondrial” axis as the central pathological hub of CIRI. Other reviews, while addressing the neuroprotective effects of MSCs in CIRI, predominantly focus on a single dimension, such as immunomodulation or mitochondrial protection, lacking an integrated analysis of how MSCs synergistically improve mitochondrial function and remodel the immune network. Furthermore, current studies present a fragmented explanation of the therapeutic mechanisms of MSCs. They do not highlight the temporal therapeutic characteristics of MSCs, namely their ability to dynamically adjust regulatory strategies according to different pathological stages of CIRI. There is also a lack of systematic understanding of novel MSC mechanisms, such as mitochondrial transfer and immunometabolic reprogramming. Most importantly, the core value of timing-based interventions targeting the “immune-mitochondrial” axis in CIRI treatment has not been clearly defined.

MSCs and their derivatives offer a novel therapeutic strategy for CIRI due to their unique temporally regulated capabilities. MSCs are a type of adult stem cells with multidirectional differentiation potential, which can be isolated from various tissues such as bone marrow, adipose tissue, and umbilical cord. Their mechanisms of action primarily involve extensive paracrine activity and immunomodulatory functions. Numerous preclinical studies have demonstrated that MSC transplantation exerts significant protective effects against CIRI [28]. Following transplantation, MSCs can migrate specifically to the site of injury and dynamically secrete various bioactive factors in accordance with different stages of the disease, exhibiting a precise temporal regulation pattern. During the acute phase of reperfusion, MSCs directly ameliorate neuronal energy metabolism and suppress oxidative stress through mitochondrial transfer or the secretion of antioxidants, thereby reducing ROS levels and inhibiting mPTP opening [29, 30]. This alleviates oxidative stress and cellular apoptosis. In the subacute phase, the regulatory focus shifts toward broad immunomodulation and pro-repair processes. MSCs secrete neurotrophic factors (such as BDNF, GDNF, NGF), anti-inflammatory cytokines (such as IL-10, TGF-β), and exosomes, among other substances [31, 32], which collectively modulate the local microenvironment. These factors can suppress the overactivation of microglia and peripheral immune cells, mitigate inflammatory responses, enhance the survival of endogenous neural cells, and promote angiogenesis and neurogenesis, thereby creating favorable conditions for tissue repair.

MSCs can differentiate into multiple cell lineages and modulate the immune microenvironment, primarily through gene regulation, anti-inflammatory responses, and regulation of various immune cells such as macrophages, T cells, and B cells, thereby promoting tissue regeneration and repair [33–35]. MSCs have also demonstrated significant efficacy in clinical trials for graft-versus-host disease [36]. Exosomes derived from MSCs can modulate the phenotype and function of immune cells, influence cellular homing, and contribute to the reprogramming of immune responses, thereby facilitating neural repair and functional recovery after cerebral ischemia-reperfusion injury [37–39].

Compared to conventional single-target drugs, MSC-based therapy exhibits broader application prospects due to its ability to coordinately regulate multiple core pathological processes, including immune inflammation, mitochondrial function, and apoptosis. Current research is progressively advancing from experimental studies to clinical practice, with several early-phase clinical trials preliminarily confirming its safety and feasibility. However, to achieve widespread clinical application, future efforts should focus on addressing key issues such as determining the optimal therapeutic time window at different pathological stages, optimizing critical parameters including cell source, infusion route, and dosage, and translating this “temporally-regulated therapeutic” strategy into tangible clinical benefits.

## Immunity and mitochondrial pathology in CIRI

### Acute stage: pathological initiation

The process of cerebral ischemia-reperfusion rapidly triggers a robust immune and inflammatory response, characterized by a distinct temporal sequence that disrupts the pre-existing immune equilibrium within the central nervous system. During the acute phase of reperfusion (within hours), this imbalance primarily manifests as the rapid activation and polarization imbalance of microglia. Subsequently, in the subacute phase (hours to days), it is characterized by the infiltration of peripheral immune cells and aberrant activation of adaptive immunity [40, 41].

In the acute phase, resident microglia, serving as the primary effector cells of innate immunity, are swiftly mobilized. They transition from their resting ramified morphology to an activated ameboid shape, resulting in dysregulation of their polarization state [42, 43]. Among these, classically activated M1-phenotype microglia are extensively activated, releasing neurotoxic substances and pro-inflammatory mediators-including tumor necrosis factor-α (TNF-α), interleukin-1β (IL-1β), ROS, and nitric oxide (NO)-which directly damage neurons and disrupt blood-brain barrier integrity [44–46]. Conversely, the alternatively activated M2-phenotype microglia, which possess reparative functions, are significantly suppressed. Their production of anti-inflammatory factors such as interleukin-10 (IL-10) and transforming growth factor-β (TGF-β) is markedly reduced, thereby inadequately counteracting the damage mediated by the M1 phenotype [47, 48]. This polarization imbalance can be regarded as a central mechanism for the initiation and persistent exacerbation of early neuroinflammation.

On the other hand, blood-brain barrier dysfunction occurring in the acute phase facilitates the entry of peripheral immune cells into the brain tissue during the subacute phase. Neutrophils, as the first wave of infiltrating inflammatory cells, exacerbate vascular endothelial and parenchymal damage through the release of proteases, ROS, and the formation of neutrophil extracellular traps (NETs) [49–51]. Subsequently, monocytes/macrophages, T cells, and B cells also migrate to the ischemic area. Among CD4⁺ T cells, pro-inflammatory subsets Th1 and Th17 amplify the inflammatory cascade by producing cytokines such as interferon-γ (IFN-γ) and interleukin-17 (IL-17) [52, 53]. In contrast, the suppressive function of regulatory T cells (Tregs) is relatively insufficient to effectively control inflammation [54, 55]. Concurrently, astrocytes and microglia upregulate the expression of MHC-II molecules and co-stimulatory molecules, acting as antigen-presenting cells to activate infiltrating T cells and establish a positive feedback loop that continuously aggravates immunopathological injury [56, 57]. This state, mediated by both innate and adaptive immunity and marked by a severe imbalance between pro-inflammatory and anti-inflammatory responses, constitutes the cornerstone of the immunopathological process during the subacute phase of cerebral ischemia-reperfusion injury.

### Subacute stage: injury expansion

The early mitochondrial dysfunction initiated by acute immune activation in the preceding phase was not effectively reversed during the reperfusion process. Its persistent deterioration became a key downstream signal for the further amplification of immune-inflammatory responses in the subacute phase, thereby forming a vicious cycle of the “immune-mitochondrial” axis. Mitochondrial damage-associated molecular patterns, such as mitochondrial DNA and formyl peptides, released by damaged mitochondria, combined with the persistent energy crisis and apoptotic signals in neurons, further activated microglia in a hyper-reactive state. This promoted the massive infiltration of peripheral immune cells, including monocytes/macrophages and T cells, into the brain parenchyma and exacerbated the abnormal activation of adaptive immunity. On this basis, mitochondrial dysfunction itself was further aggravated during the subacute phase, becoming a core pathological driver of injury amplification.

Mitochondria, as the central organelles for intracellular energy production and metabolic regulation, are highly sensitive to hypoxic conditions and represent the most vulnerable components in CIRI. Their dysfunction persists throughout the acute phase and drives subsequent damage.

Although reperfusion restores oxygen supply, it fails to recover mitochondrial function and instead induces more pronounced dysfunction, a phenomenon termed the “mitochondrial paradox” [58]. This paradox is initially manifested as severe disruption of energy metabolism. During the ischemic phase, the deficiency of oxygen and glucose halts OXPHOS, leading to a rapid decline in ATP levels [59]. Despite the renewed availability of oxygen in the acute phase, functional units such as complex I of the electron transport chain (ETC), damaged during hypoxia, result in electron leakage. These electrons combine with oxygen to generate excessive ROS, primarily superoxide anions, thereby triggering oxidative stress [60]. High concentrations of ROS not only cause direct damage to mitochondrial DNA, membrane lipids, and proteins but also further inhibit ETC activity, exacerbating the impairment of energy synthesis. Consequently, the prolonged insufficiency of ATP regeneration compromises cellular repair mechanisms, ultimately leading to ionic homeostasis imbalance, edema, and cell death [61, 62].

Another critical mechanism occurring in the acute phase is the pathological opening of the mPTP. Activated by factors such as calcium overload, elevated ROS levels, and increased phosphate concentrations, the sustained opening of mPTP causes the collapse of the mitochondrial membrane potential, completely abrogates ATP synthesis, and induces mitochondrial swelling and outer membrane rupture. This leads to the release of apoptosis-related factors, such as cytochrome c (Cyt c), into the cytoplasm [63]. In the cytosol, Cyt c assembles with Apaf-1 to form the apoptosome, which sequentially activates caspase-9 and caspase-3, thereby initiating the apoptosis program [64, 65]. Thus, mPTP is considered a key molecular switch linking ischemic injury to programmed cell death during the acute phase of reperfusion.

In addition, the imbalance in mitochondrial dynamics, including disturbances in fusion and fission processes, and impaired mitophagy, collectively contribute to the progression of reperfusion injury. Ischemia-reperfusion enhances Drp1-mediated mitochondrial fission, resulting in the accumulation of numerous fragmented and dysfunctional mitochondria [66, 67]. Concurrently, autophagy pathways responsible for clearing damaged mitochondria, such as the PINK1/Parkin pathway, are often inefficient or overloaded, preventing the effective degradation of dysfunctional mitochondria and further exacerbating oxidative stress and the risk of apoptosis [68].

### Interplay between the immune system and mitochondria

The above pathological changes in the acute and subacute phases fully demonstrate the bidirectional and sequential regulatory relationship of the “immune-mitochondrial” axis in CIRI: immune activation is the upstream initiator of mitochondrial dysfunction, and the subsequent deterioration of mitochondrial damage is the downstream amplifier of immune inflammation. Their mutual promotion and cycle formation constitute the core pathological basis of CIRI injury progression, and the specific molecular interplay mechanisms between the immune system and mitochondria are elaborated as follows:

The immune response and mitochondrial dysfunction are not independent pathological processes; rather, they engage in a close, bidirectional, and temporally sequential interaction. This interplay persists across different stages of CIRI, constituting a vicious cycle of the “immune-mitochondrial” axis. Mitochondria serve as central hubs for metabolism and signal transduction within immune cells, significantly influencing cell fate decisions and functional states, thereby participating in the regulation of inflammatory response progression during CIRI.

The polarization direction of microglia is directly regulated by mitochondrial function, a process closely associated with their metabolic reprogramming. Classically activated M1 phenotypes exhibit pro-inflammatory properties and primarily rely on glycolysis for rapid ATP generation to meet the energy and biosynthetic demands required for the high-intensity synthesis of inflammatory mediators [68]. This metabolic shift is regulated by hypoxia-inducible factor-1α (HIF-1α), whose stability is positively modulated by mitochondrial ROS [69]. Conversely, alternatively activated M2 phenotypes demonstrate anti-inflammatory and reparative functions, with their energy supply being more dependent on OXPHOS [70]. In the context of CIRI, impaired mitochondrial OXPHOS capacity hinders M2 polarization at the metabolic level, while excessive ROS production promotes M1 polarization. This ultimately leads to an imbalance in microglial phenotypes and exacerbates neuroinflammation.

The differentiation and activation of T cells infiltrating the central nervous system are also precisely regulated by the mitochondrial metabolic state. Upon activation, naive T cells undergo a dramatic metabolic transition, shifting from OXPHOS dependence in the resting state to active glycolysis to support their clonal expansion and effector functions [71, 72], a process analogous to that observed in M1 macrophages/microglia. Mitochondria-derived ROS can act as second messengers to activate signaling pathways such as NF-κB, promoting T cell proliferation and the release of pro-inflammatory cytokines like interferon-gamma (IFN-γ) and interleukin-2 (IL-2) [73]. Furthermore, different T cell subsets exhibit preferences for distinct metabolic pathways: the pro-inflammatory Th17 subset relies on glycolysis, whereas the differentiation of Tregs requires OXPHOS and fatty acid oxidation (FAO) support [74]. Mitochondrial dysfunction, ROS accumulation, and impaired OXPHOS present in CIRI collectively shape a metabolic microenvironment that favors Th1/Th17 differentiation while suppressing Tregs, thereby skewing the adaptive immune response toward a pro-inflammatory direction.

Additionally, in the acute phase, when severe damage occurs in neurons or glial cells, mitochondria can release their contents into the extracellular space. These components act as DAMPs and activate innate immune responses by engaging pattern recognition receptors (PRRs) on the surface of immune cells [75]. For instance, mitochondrial DNA (mtDNA), which contains unmethylated CpG motifs similar to bacterial DNA, can be recognized by Toll-like receptor 9 (TLR9); mitochondrial formyl peptides can bind formyl peptide receptors [76]; and released ATP can activate the purinergic receptor P2X7 [77]. These mitochondria-derived DAMPs potently activate microglia and other immune cells, promoting the assembly and activation of the NLRP3 inflammasome. This, in turn, induces the maturation and release of key inflammatory cytokines such as interleukin-1β (IL-1β) and IL-18, forming a positive feedback loop from acute mitochondrial damage to subacute excessive immune activation.

**Fig. 1:**
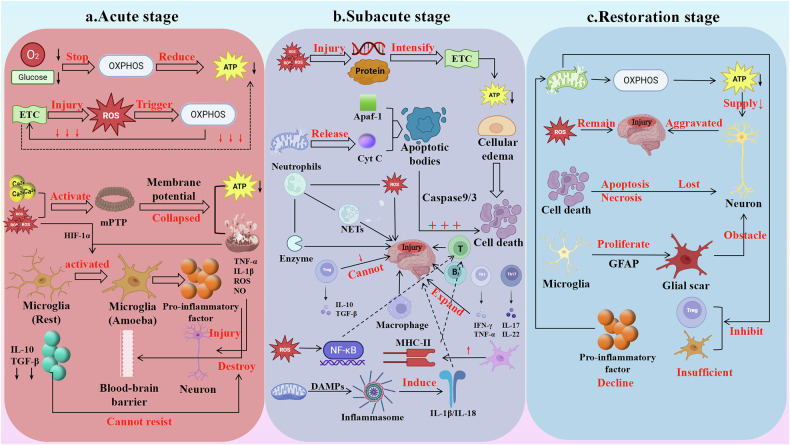
Pathological mechanisms of CIRI at different stages. **a** Acute stage: Mitochondrial dysfunction is initiated and exacerbated, leading to an oxidative stress burst. The pathological opening of the mPTP occurs, accompanied by an imbalance in mitochondrial dynamics. Innate immunity is rapidly activated, disrupting the immune balance, primarily characterized by an imbalance in microglial polarization. Initial dysfunction of the blood-brain barrier is observed. **b** Subacute stage: Mitochondrial dysfunction persists, with cumulative oxidative stress and mitochondrial damage. Apoptotic pathways are initiated and amplified. Peripheral immune cells infiltrate the brain, and adaptive immunity is aberrantly activated. A positive feedback loop forms between innate and adaptive immunity, amplifying the inflammatory response and exacerbating the imbalance between pro-inflammatory and anti-inflammatory signals. **c** Restoration stage: Mitochondrial function fails to fully recover, resulting in the accumulation of damaged mitochondria. Cell death continues, leading to irreversible brain tissue damage, including neuronal loss and glial scar formation. While the inflammatory response gradually resolves, tissue healing and immunoregulatory functions remain impaired long-term, potentially resulting in a state of chronic inflammation or immunosuppression.

Currently, there is no unified and systematic authoritative definition for the disease stages and temporal scope of CIRI, and dedicated research findings in this area are relatively scarce. Only a few publications sporadically mention the core characteristics and partial connotations of this concept in related discussions [78]. Based on summarizing and refining the common understandings of this concept found in the limited representative literature, and considering the core requirements of this study, this review aims to provide a targeted definition of the connotations and boundaries of the disease stages and temporal scope of CIRI. This is intended to establish a unified cognitive baseline for subsequent research and analysis. Therefore, Fig. 1 was created to illustrate the different pathological mechanisms across the three distinct stages of CIRI, while Table 1 was constructed to depict the interactions and influences between immune cells and mitochondria in CIRI.

**Table 1:** Interactions between immune cells and mitochondria in CIRI and their effects.

Cell type	Interaction mechanisms	Effects	References
Microglia	M1 macrophages rely on glycolysis, whereas M2 macrophages depend on oxidative phosphorylation. Mitochondrial dysfunction releases components such as mtDNA, which activates immune responses, and ROS that amplify inflammation. The clearance of damaged mitochondria via mitophagy can attenuate inflammation. Meanwhile, MSCs can improve macrophage function through mechanisms including mitochondrial transfer.	Mitochondrial dysfunction disrupts immune homeostasis and exacerbates neuroinflammation, whereas its restoration facilitates the reestablishment of immune homeostasis, thereby effectively mitigating inflammatory damage.	Wang et al. [68] Tabima et al. [69] Sonia Missiroli et al. [75] Wanka et al. [108]
Neutrophils	As the first peripheral immune cells to infiltrate, they function through the release of proteases, ROS, and the formation of NETs.	They exacerbate damage to the vascular endothelium and brain parenchyma, amplifying the inflammatory response.	Sánchez-Hernández et al. [49]
Dendritic Cells	Following ischemic stroke, they are activated, and their temporal dynamics, activation status, and origin undergo changes.	As professional antigen-presenting cells, they bridge innate and adaptive immunity, potentially initiating and amplifying T cell-mediated immune responses.	Felger et al. [27]
Monocytes/Macrophages	Metabolites such as succinate influence the expression of genes like Foxp3 through histone modifications (e.g., succinylation).	They affect the stability of Tregs and the polarization direction of macrophages.	Mulik et al. [134]
T Lymphocytes (Th1/Th17, Treg)	Effector T cells rely on glycolysis, whereas Tregs depend on oxidative phosphorylation and fatty acid oxidation. Mitochondrial ROS activate NF-κB to promote the differentiation of Th1and Th17 cells. Metabolites such as succinate regulate the expression of Foxp3 through epigenetic modifications.	Enhancing pro-inflammatory T cell responses and cytokine release while suppressing Treg function, or promoting macrophage M2 polarization, reducing neutrophil infiltration, and enhancing Treg function.	Cattani-Cavalieri et al. [73] Simon-Molas et al. [74] Mulik et al. [134] Hui et al. [136] Jiang et al. [113]
B Lymphocytes	They migrate from the periphery to the lesion sites in the brain tissue.	As adaptive immune cells, they participate in and exacerbate inflammation.	AlRuwaili et al. [17]
Astrocytes	Upon activation, they upregulate the expression of MHC-II molecules and co-stimulatory molecules, functioning as antigen-presenting cells.	They activate infiltrating T lymphocytes, forming a positive feedback loop that aggravates immunopathological damage.	Giménez-Orenga et al. [56]

## Mitochondrial protection and functional enhancement by MSCs and their derivatives

MSCs directly target mitochondrial functional repair through synergistic multi-pathway actions. They not only directly provide functional mitochondria to damaged cells via tunneling nanotubules but also regulate mitochondrial dynamics, enhance the antioxidant defense system, promote biosynthesis, and remodel energy metabolism patterns through the secretion of bioactive substances.

### Acute stage rescue: direct mitochondrial transfer

MSCs can actively transfer mitochondria to damaged cells, a behavior considered a key mechanism mediating their cytoprotective effects, particularly significant during the acute phase requiring rapid energy replenishment. This transfer process can be achieved via various intercellular connections, such as tunneling nanotubules, exosomes, or other microvesicle-mediated contact pathways [79]. In various disease models, including acute lung injury, myocardial ischemia, and neurotoxicity, MSCs have been observed to directly deliver functional mitochondria to damaged epithelial cells, cardiomyocytes, or neurons through tunneling nanotubules (TNTs), thereby enhancing their ATP production capacity, promoting cell survival, and inhibiting apoptosis [80]. Stress signals associated with CIRI upregulate adhesion molecules and cytoskeletal proteins, thereby driving the assembly of F-actin-dependent TNTs. Mitochondrial dysfunction in recipient cells activates the PI3K/Akt/mTOR pathway in MSCs, which mediates the directional transfer of mitochondria [81–83]. Experiments on alveolar epithelial injury have also confirmed that mitochondrial transfer from MSCs significantly enhances the metabolic function of epithelial cells [84]. The successful implementation of this mechanism relies on membrane connections formed between MSCs and target cells and the remodeling of the actin cytoskeleton. On the other hand, exosomes secreted by MSCs also contain mitochondrial components or intact mitochondrial DNA, which can be taken up by recipient cells and integrated into the existing mitochondrial system, providing energy support and genetic repair indirectly [85].

In addition to the direct transfer of intact mitochondria via tunneling nanotubes or exosomes, the apoptosis of systemically administered MSCs in the harsh pathological microenvironment of CIRI also serves as an important active regulatory mechanism for mitochondrial protection in the acute phase, and this process plays a critical role in the therapeutic efficacy of MSCs. Apoptotic MSCs release apoptotic bodies rich in mitochondrial bioactive components, including mitochondrial‑derived peptides, functional mitochondrial membrane protein subunits, and mitochondrial DNA fragments. These components can be phagocytosed by damaged neurons and glial cells in the lesion area, directly supplementing defective mitochondrial functional machinery, activating the AMPK/PGC‑1α pathway to promote mitochondrial biogenesis, and restoring mitochondrial electron transport chain activity to reduce reactive oxygen species burst [86–88]. Meanwhile, the mild apoptotic signals from MSCs trigger a controlled innate immune response, promoting the polarization of microglia toward the M2 phenotype and enhancing the phagocytic clearance capacity for damaged mitochondria in the lesion area, thereby further blocking the oxidative stress and inflammatory pathological cascade triggered by mitochondrial dysfunction [89, 90]. This apoptosis-mediated mitochondrial protection mode, in synergy with direct mitochondrial transfer, constitutes a dual compensatory mechanism through which MSCs mediate the rescue of mitochondrial function in acute-phase CIRI.

MSCs do not ubiquitously transfer mitochondria. Instead, they achieve targeted transfer to damaged immune cells by recognizing pathological stress signals, such as TNF-α and DAMPs, released by these cells (e.g., M1-type microglia and Th1/Th17 cells). This process is facilitated by the specific binding between CD73/CD105 on the MSC surface and CD44/ICAM-1 on the surface of the recipient cells [91–93]. Following transfer, the functional mitochondria enter the recipient cells and fuse with the endogenous mitochondrial network via fusion proteins such as Mfn1/2 and Opa1. This fusion subsequently restores oxidative phosphorylation activity and ATP synthesis in the recipient cells, reduces mitochondrial reactive oxygen species production, and further modulates the metabolic reprogramming of the immune cells, thereby reversing their pro-inflammatory phenotype [86, 87, 94]. In allogeneic MSC therapy, the donor mtDNA transferred synchronously with the mitochondria rarely induces immune rejection. This is primarily because the donor mtDNA is mostly encapsulated within mitochondrial fragments or exosomes, which avoids activation of the innate immune response. Concurrently, MSCs can establish a local immune-tolerant microenvironment by inhibiting the activation of host immune cells. Furthermore, the low copy number of the transferred donor mtDNA is insufficient to trigger immune recognition by the host [95, 96].

### Subacute stage modulation of mitochondrial dynamics

Mitochondrial dynamics, referring to the balance between mitochondrial fusion and fission, is crucial for maintaining the functional integrity of the mitochondrial network and cellular homeostasis. During the acute phase of CIRI, excessive fission often leads to mitochondrial fragmentation, dysfunction, and apoptosis [97], whereas the fusion process helps complement contents and stabilize membrane potential [98]. MSCs and their secreted products can regulate the expression of proteins related to mitochondrial dynamics by releasing various bioactive molecules, including growth factors, cytokines, and non-coding RNAs. Specifically, hepatocyte growth factor (HGF) and vascular endothelial growth factor (VEGF), abundant in MSC-conditioned medium, can promote the upregulation of fusion proteins (Mfn1, Mfn2, and Opa1) and inhibit the activity of fission proteins (Drp1, Fis1, and MPT18) [99]. In doxorubicin-induced cardiomyopathy, miR-125b-5p carried by MSC-derived exosomes inhibits Drp1, thereby attenuating mitochondrial fission and improving myocardial contractile function [100]. Similarly, in the context of neurodegenerative diseases, MSC-derived exosomes can alleviate excessively activated mitophagy by regulating the PINK1/Parkin pathway, thereby protecting neurons from CIRI damage [68]. By remodeling the balance of mitochondrial dynamics, MSCs help restore the health and stability of the mitochondrial network during the acute phase.

### Subacute stage reduction of oxidative stress

Oxidative stress, a core aspect of mitochondrial dysfunction in the acute phase, induces substantial accumulation of mitochondrial ROS, subsequently damaging its DNA, lipids, and protein structures [101]. MSCs not only possess a strong capacity to resist oxidative damage themselves but also enhance the antioxidant defense system of recipient cells through paracrine mechanisms. They can secrete enzymatic substances such as superoxide dismutase (SOD), catalase (CAT), and glutathione peroxidase (GPx) to directly neutralize ROS [102]. Meanwhile, exosomal miR-155 suppresses the expression of antioxidant-related genes and increases ROS levels by impairing tolerance to oxidative stress, while miR-142 induces ROS accumulation by inhibiting heterophagy in senescent bone marrow MSCs [103]. It has been reported that MSC-conditioned medium can enhance the activities of mitochondrial complexes I and III, reduce electron leakage, thereby suppressing ROS production at the source [104]. This indicates that MSCs can implement effective interventions during the acute phase of oxidative stress outburst from both the “clearance” and “source inhibition” perspectives.

### Subacute stage promotion of mitochondrial biogenesis

In addition to directly protecting mitochondria, MSCs can promote mitochondrial biogenesis in recipient cells. This process is primarily mediated via the peroxisome proliferator-activated receptor γ coactivator-1α (PGC-1α) signaling pathway [105]. Various factors secreted by MSCs, such as insulin-like growth factor 1 (IGF-1) and fibroblast growth factor 21 (FGF21), can upregulate the expression of PGC-1α, thereby promoting the transcription and translation of nuclear-encoded mitochondrial genes and facilitating the formation of new mitochondria [106]. For instance, studies on aging have found that MSC-conditioned medium can restore SIRT1 activity in senescent cells, enhance the deacetylation level of PGC-1α, thereby reversing the decline in mitochondrial biogenesis capacity [107]. Although mitochondrial biogenesis is a relatively late process, its successful initiation relies on the stabilization of the acute phase environment. MSCs create conditions for cells to initiate repair programs through acute phase interventions, thereby supporting energy metabolism recovery in the subacute and recovery phases.

### Recovery stage improvement of mitochondrial metabolism and energy supply

MSCs and their derivatives can also enhance mitochondrial energy output by remodeling cellular metabolic patterns. Under stress conditions, cells often exhibit enhanced glycolysis and weakened oxidative phosphorylation, known as the “Warburg effect” [108]. MSCs can regulate key metabolic enzymes and mitochondrial carriers, prompting cells to shift towards more efficient oxidative metabolism. For example, in the tumor microenvironment or local ischemic tissues, miR-122 in MSC-derived exosomes can inhibit the expression of pyruvate kinase M2 (PKM2), promote glucose entry into the tricarboxylic acid (TCA) cycle, and improve ATP production efficiency [109]. Furthermore, metabolic substrates such as lactate and ketone bodies contained in MSC-conditioned medium can also be utilized by mitochondria as alternative energy sources, particularly under pathological conditions with insufficient glucose supply [110]. This metabolic remodeling function is crucial for helping cells survive the energy crisis of the acute phase and laying the metabolic foundation for subsequent repair. In our previous animal studies [111], it was found that MSCs and their derivatives could significantly alleviate oxidative stress-induced neuronal damage by upregulating the expression of SIRT1 and improve cognitive function in aging models. This finding fills the mechanistic gap regarding how MSCs regulate neuronal metabolism and stress responses via the SIRT1-PGC-1α axis, providing direct experimental evidence for understanding how stem cells achieve neuroprotection at the metabolic level. As a core regulator of energy metabolism and cellular stress response, SIRT1 plays a key role in maintaining neuronal homeostasis. It fundamentally ameliorates the energy metabolic crisis and oxidative damage in neurons, serving as a critical link connecting stem cell therapy with the metabolic remodeling of neural cells.

MSCs and their derivatives effectively target the core pathological link of mitochondrial dysfunction during the critical time window after CIRI, especially in the acute phase, laying a solid foundation for cell survival and subsequent immune network remodeling. The pathological process of CIRI is not static but evolves according to strict temporal sequence patterns. The excellence of MSC therapy lies in the dynamic adaptation of its mechanisms of action to the pathological evolution of CIRI, allowing for precise regulation of temporal changes. Based on the dynamic regulation by MSCs, therapeutic strategies for different time windows of CIRI are summarized in Table 2.

**Table 2:** Therapeutic strategies for CIRI at different time windows.

Disease stage	Time range	Pathological features	Mechanisms of action	Therapeutic goals	References
Acute stage	Minutes to hours	Energy depletion, oxidative stress, and apoptosis are the core pathological processes of mitochondrial dysfunction, collectively exacerbating cellular damage.	By transferring mitochondrial energy, enhancing antioxidant capacity, and regulating dynamics, cellular function is comprehensively improved.	Objective: Rescue the ischemic penumbra and maintain basic neuronal viability. Strategy: Rapid intervention to provide direct life support	Hou et al. [101] García et al. [137]
Subacute stage	Hours to Days	Excessive activation of the innate immune system triggers neuroinflammation, which is characterized by immune cell infiltration and a cytokine storm.	They exert therapeutic effects by improving mitochondrial function (promoting oxidative phosphorylation and reducing reactive oxygen species (ROS)), modulating immunity (promoting M2 polarization and inhibiting the NLRP3 inflammasome), and secreting anti-inflammatory factors.	Objective: To control the scale of inflammation, prevent the expansion of injury, and protect the blood-brain barrier. Strategy: Systemic immunomodulation to quell the inflammatory storm, thereby creating a prerequisite for repair.	Ye et al. [138] Doblado et al. [139] Anamika et al. [122]
Restoration stage	Days to Weeks	Adaptive immune dysregulation is initiated, accompanied by persistent metabolic disturbances.	Metabolic reprogramming and mitochondrial biogenesis suppress pathogenic T cells and enhance regulatory T cell (Treg) function, while the secretion of reparative factors and modulation of glial cells synergistically promote neural repair and immune homeostasis.	Objective: To establish immune tolerance and promote neurovascular repair and functional remodeling. Strategy: Long-term support and reconstruction to guide the organism from a destructive state towards repair.	Zhao et al. [52] Wang et al. [140] Okada et al. [107]

## Immune network remodeling driven by improved mitochondrial function

The restoration of mitochondrial function by MSCs and their derivatives during the acute phase of reperfusion not only directly protects neurons but also creates a prerequisite for the systematic remodeling of the imbalanced immune network during the subacute and recovery phases. The improved cellular metabolism and energy homeostasis remodel surrounding immune cells from a pro-inflammatory phenotype to an anti-inflammatory phenotype through various mechanisms, thereby breaking the vicious cycle of the “immune-mitochondria” axis. Our previous study demonstrated that in a periodontitis model [112], MSCs and their derivatives can directly regulate the energy metabolism and redox status of recipient cells through the key mechanism of “mitochondrial transfer,” effectively reshaping the local immune microenvironment. This provides a new perspective for understanding the dual “metabolic–immune” regulatory mechanism of MSCs in inflammatory diseases and offers cross-disease evidence supporting dynamic immunomodulatory strategies in CIRI treatment.

### Regulation of innate immunity in the acute/subacute phase

Microglia and macrophages, as crucial components of the innate immune system, exhibit high plasticity and can polarize into pro-inflammatory (M1) or anti-inflammatory (M2) phenotypes in response to microenvironmental signals. Mitochondrial function plays a central regulatory role in this polarization process. Studies indicate that mitochondrial metabolic reprogramming underlies macrophage polarization, where M1 polarization relies on glycolysis, while M2 polarization depends on oxidative phosphorylation (OXPHOS). MSCs improve mitochondrial function by enhancing electron transport chain complex activity and reducing mitochondrial reactive oxygen species (mtROS), thereby promoting the polarization of microglia/macrophages towards the M2 phenotype during the immunologically active subacute phase, consequently enhancing their anti-inflammatory and tissue healing capabilities [113]. For instance, mitochondrial-derived peptides Humanin and MOTS-c can enhance mitochondrial biogenesis and fatty acid oxidation by modulating the AMPK/PGC-1α signaling pathway, subsequently inducing M2 macrophage polarization [114]. Furthermore, moderate regulation of mtROS can also suppress excessive activation of NF-κB, reducing the release of pro-inflammatory factors such as TNF-α and IL-1β, and further promoting the shift of microglia/macrophages towards an anti-inflammatory phenotype [115]. This signifies a transition in the role of MSCs from intrinsic cellular protection in the acute phase to local microenvironment immune regulation in the subacute phase.

The NLRP3 inflammasome is a key multiprotein complex in innate immunity, whose activation leads to caspase-1-mediated maturation of IL-1β and IL-18, triggering a robust inflammatory response [116]. Mitochondria play a significant role in NLRP3 activation, where mtROS, mitochondrial DNA (mtDNA) release, and collapse of the mitochondrial membrane potential can directly activate NLRP3 [117]. By reducing mtROS production and enhancing mitophagy to clear damaged mitochondria during the acute phase, MSCs can effectively prevent the release of mitochondrial-associated damage-associated molecular patterns (DAMPs) like mtDNA, thereby blocking the assembly and activation of NLRP3 at the critical stage of inflammasome activation [118–120]. This mechanism severs a crucial pathway where mitochondrial damage directly triggers intense inflammation, serving as a bridge connecting mitochondrial protection and innate immune regulation. Our previous study confirmed that MSCs-derived exosomes, in synergy with other active components, can effectively promote the polarization of macrophages from the pro-inflammatory M1 phenotype to the reparative M2 phenotype. Simultaneously, they block pyroptosis by inhibiting the NLRP3 inflammasome pathway, thereby exerting potent anti-inflammatory and tissue-protective effects in an arthritis model. This provides a combinatorial strategy for the development of MSCs-based “multi-target immune remodeling therapy,” advancing the application prospects of cell-free therapies in neuroinflammatory diseases [121]. This suggests that in CIRI, MSCs reshape the microglia/macrophage-mediated immune network through a dual mechanism of “modulating polarization and inhibiting pyroptosis,” thereby creating favorable conditions for neural repair.

### Containment of subacute phase inflammatory burst: inhibition of neutrophil infiltration and NETs formation

Neutrophils are key effector cells in acute inflammatory responses, and their excessive activation and infiltration can lead to tissue damage. The formation of NETs is a specific immune function of neutrophils, but their excessive formation is associated with various autoimmune and inflammatory diseases. Mitochondrial function regulation can influence neutrophil behavior through multiple pathways. Firstly, mtROS is a key promoter of neutrophil activation and NETosis [122]. By improving mitochondrial metabolism and reducing ROS bursts, neutrophil migration and activation can be significantly suppressed [123]. Secondly, maintaining mitochondrial calcium homeostasis can reduce neutrophil chemotaxis and degranulation [124]. Some studies suggest that enhancing mitochondrial biogenesis can reduce neutrophil retention and NETs release at inflammatory sites by modulating glycolytic preference [125]. This indicates that the antioxidant and metabolic homeostasis established by MSCs during the acute phase can effectively curb the explosive damage caused by neutrophils in the subacute phase. In our previous studies on systemic inflammatory models such as disseminated intravascular coagulation [126], MSC-Exos demonstrate potent systemic immunomodulatory and homeostatic remodeling capabilities. They can systematically correct dysregulated immunocoagulatory responses by modulating M1/M2 macrophage polarization, inhibiting the formation of NETs, and balancing pro-inflammatory and anticoagulant pathways. This study is the first to demonstrate in a critical systemic inflammation model that MSC-derived products can coordinately restore homeostasis in the two closely linked pathological processes-immune response and coagulation function-through synchronous multi-target intervention. This provides a crucial theoretical extension and strategic reference for their application in localized inflammatory diseases accompanied by microvascular coagulopathy, such as CIRI. MSCs and their derivatives simultaneously target microglial/macrophage phenotypic transition, suppress neutrophil-mediated inflammatory injury, and improve local microvascular coagulopathy, thereby achieving holistic remodeling and protection of the neuroimmune microenvironment.

### Recovery stage suppression of pathogenic T cell responses

During the subacute and recovery phases of reperfusion, the excessive activation of pathogenic T cells (e.g., Th1, Th17) in the adaptive immune response is a hallmark of various autoimmune diseases. T cell differentiation and function are highly dependent on metabolic reprogramming; Teff rely on glycolysis, whereas Tregs depend on OXPHOS and FAO [127]. Improved mitochondrial function can suppress pathogenic T cell responses through various mechanisms. For example, enhanced mitochondrial oxidative metabolism can reduce the expansion of glycolysis-dependent Teff cells, while influencing transcriptional programs by modulating acetyl-CoA levels and histone acetylation [128]. Furthermore, regulation of mitochondrial membrane potential and ROS levels can alter T cell receptor (TCR) signaling strength and the activation of downstream NFAT and NF-κB pathways, thereby inhibiting the production of pro-inflammatory cytokines such as IL-17 and IFN-γ [129]. Studies have shown that mitochondrial-targeted therapies can significantly alleviate Th1/Th17 responses in experimental autoimmune encephalomyelitis (EAE) and multiple sclerosis models [130]. This demonstrates the ability of MSCs to implement precise regulation during the adaptive immune activation phase through metabolic intervention.

### Recovery stage promotion of treg generation and function

Tregs play a critical role in maintaining immune tolerance and suppressing excessive inflammation. The differentiation and function of Tregs are heavily dependent on mitochondrial metabolism, particularly mitochondrial FAO and OXPHOS [131]. Improved mitochondrial function can directly promote Treg generation and stability. For instance, AMPK activators and PGC-1α agonists can enhance mitochondrial biogenesis and FAO, thereby supporting the metabolic demands of Tregs and enhancing their suppressive function [132]. Mitochondrial dynamics also influence Treg function; overexpression of the mitochondrial fusion protein Mitofusin 2 (Mfn2) can enhance the immunosuppressive capacity of Tregs [133]. Additionally, mitochondrial metabolites (e.g., succinate) can regulate Foxp3 expression and Treg stability through epigenetic modifications such as histone succinylation [134]. During the recovery phase, promoting Treg function is crucial for terminating inflammation, establishing immune tolerance, and initiating the repair stage; MSCs play a promotive role in this process by providing necessary metabolic support.

### Recovery stage modulation of astrocyte immunoregulatory functions

Astrocytes are important immunoregulatory cells in the central nervous system, influencing neuroinflammation by releasing cytokines and chemokines. Mitochondrial dysfunction is closely associated with aberrant astrocyte activation and inflammatory phenotypes. Improving mitochondrial function can modulate the immunoregulatory properties of astrocytes. For example, enhanced mitochondrial metabolism can promote the production of neurotrophic factors (e.g., GDNF, BDNF) and anti-inflammatory factors (e.g., TGF-β, IL-10) by astrocytes, while reducing the release of pro-inflammatory factors [135]. Improved mitochondrial Ca²⁺ buffering capacity can also suppress excessive activation of the NF-κB and MAPK pathways. MSCs and their derivatives, through multifaceted restoration of mitochondrial function during the acute phase, sequentially and systematically shift the immune response from a destructive inflammatory storm toward a protective reparative program in the subacute phase of CIRI. Ultimately, this process achieves a synergistic effect of neuroprotection and tissue healing.

**Fig. 2:**
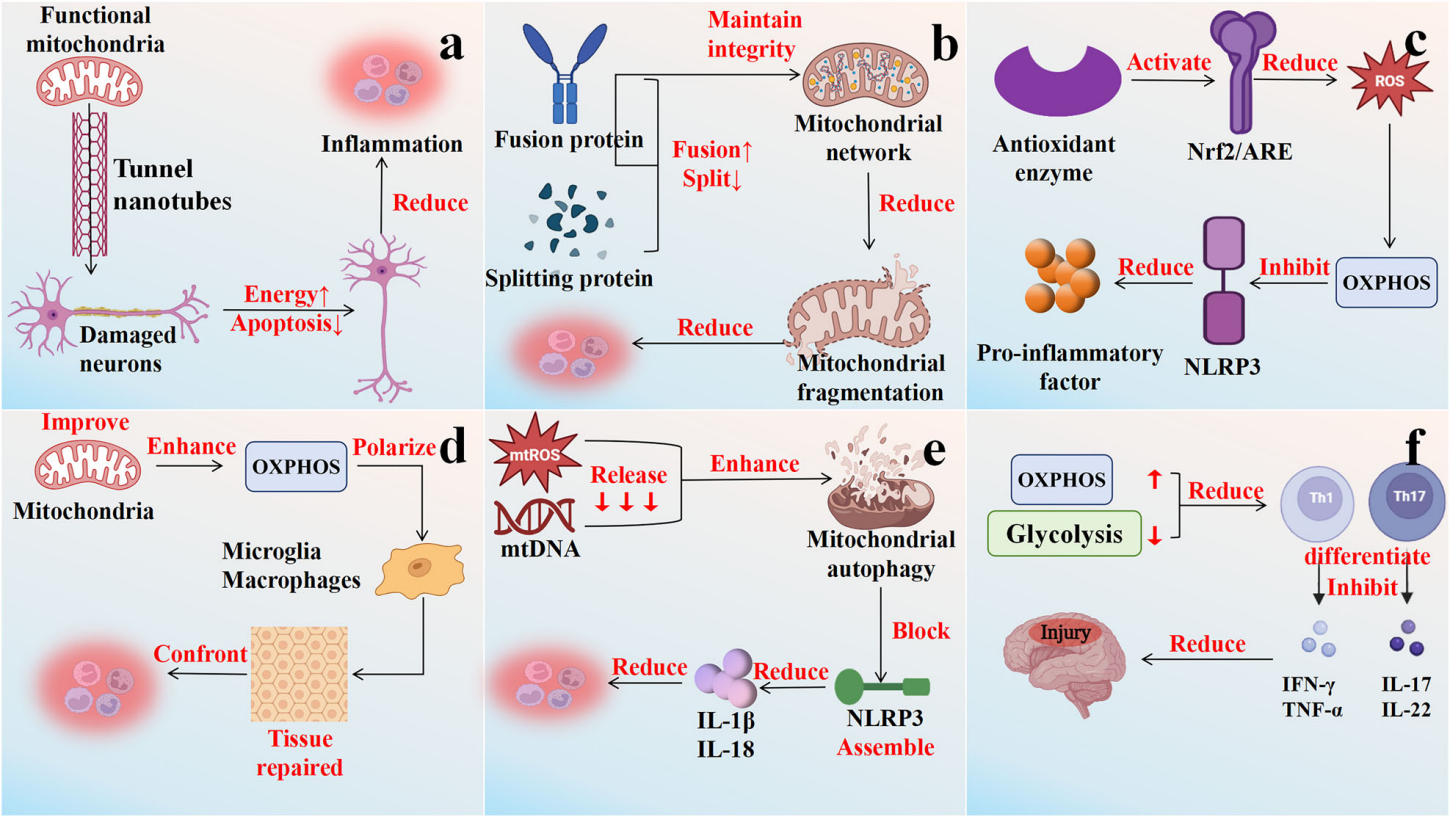
MSCs ameliorate CIRI by modulating mitochondrial remodeling and immune responses. **a** MSCs transfer functional mitochondria to damaged neurons via tunneling nanotubes, thereby enhancing cellular energy metabolism, reducing apoptosis, and indirectly attenuating immune-inflammatory responses. **b** MSCs regulate fusion and fission proteins to promote mitochondrial fusion and suppress excessive fission, maintaining mitochondrial network integrity and consequently reducing inflammation triggered by mitochondrial fragmentation. **c** MSCs secrete antioxidant enzymes and activate the Nrf2/ARE pathway, leading to decreased ROS production, reduced oxidative stress, inhibition of NLRP3 inflammasome activation, and diminished release of pro-inflammatory cytokines. **d** MSCs improve mitochondrial metabolism and promote microglial polarization toward an anti-inflammatory phenotype, thereby enhancing tissue healing capacity and suppressing neuroinflammation. **e** MSCs reduce the release of mtROS and mtDNA, enhance mitophagy, and block NLRP3 inflammasome assembly, resulting in decreased secretion of inflammatory factors such as IL-1β and IL-18, and alleviation of inflammatory injury. **f** MSCs enhance OXPHOS and suppress glycolysis, leading to reduced differentiation of Th1/Th17 cells and inhibition of pro-inflammatory cytokine release, thereby mitigating adaptive immune damage.

The core restorative effect of MSCs and their derivatives on CIRI lies in their regulation of the dynamic changes in mitochondria within different immune cells, thereby remodeling the immune response. Figure 2 and Table 3 detail the specific mechanisms and effects of this process.

**Table 3:** MSCs regulate mitochondrial remodeling and immune response to ameliorate CIRI.

Mechanism of action	Specific mode of action	Impact on the immune system	References
Direct Mitochondrial Transfer	Functional mitochondria are transferred to damaged neurons via TNTs, exosomes, and other mechanisms.	This enhances cellular energy metabolism, reduces apoptosis, and indirectly attenuates immune-mediated inflammatory responses.	Zhang et al. [80]
Regulation of Mitochondrial Dynamics	The expression of fusion proteins (Mfn1/2, Opa1) and fission proteins (Drp1) is modulated to promote mitochondrial fusion and suppress excessive fission.	This maintains mitochondrial network integrity and reduces inflammation triggered by mitochondrial fragmentation.	Yin et al. [99]
Reduction of Oxidative Stress	Secretes antioxidant enzymes (SOD, CAT, GPx), activates the Nrf2/ARE pathway, and reduces ROS production.	Decreases oxidative stress, inhibits NLRP3 inflammasome activation, and reduces the release of pro-inflammatory factors.	Pham-Huy et al. [102]
Promotion of Mitochondrial Biogenesis	Upregulates genes related to mitochondrial biogenesis via the PGC-1α signaling pathway.	Enhances cellular energy supply, supporting the function of anti-inflammatory immune cells (e.g., M2 macrophages, Treg cells).	Baskin et al. [106]
Improvement of Mitochondrial Metabolism and Energy Supply	Regulates metabolic enzymes (e.g., PKM2), promotes glucose entry into the TCA cycle, and enhances OXPHOS.	Promotes the shift of immune cells toward an anti-inflammatory phenotype and inhibits pro-inflammatory T-cell responses.	Wanka et al. [108]
Regulation of Microglial/Macrophage Polarization	Promotes M2 polarization by improving mitochondrial metabolism (e.g., enhancing OXPHOS).	Strengthens anti-inflammatory and tissue healing capacity and suppresses neuroinflammation.	Jiang et al. [113]
Inhibition of NLRP3 Inflammasome Activation	Reduces mtROS and mtDNA release, enhances mitophagy, and blocks NLRP3 assembly.	Inhibits the release of inflammatory factors such as IL-1β and IL-18, thereby mitigating inflammatory injury.	Fang et al. [116]
Suppression of Neutrophil Infiltration and NETs	Decreases ROS and calcium overload, inhibits neutrophil activation and NETosis.	Alleviates vascular endothelial and brain parenchymal damage, ameliorating acute inflammatory responses.	Anamika et al. [122]
Inhibition of Pathogenic T-Cell Responses	Reduces Th1/Th17 cell differentiation by enhancing OXPHOS and inhibiting glycolysis.	Suppresses the release of pro-inflammatory cytokines (IFN-γ, IL-17), attenuating adaptive immune injury.	Navabi et al. [129]
Promotion of Treg Generation and Function	Supports the metabolic demands of Treg cells by enhancing FAO and OXPHOS, thereby augmenting their suppressive function.	Enhances immune tolerance and curbs excessive inflammatory responses.	Li et al. [131]
Modulation of Astrocyte Function	Improves mitochondrial metabolism, promotes the release of neurotrophic factors and anti-inflammatory factors, and inhibits the NF-κB/MAPK pathway.	Regulates the neuroinflammatory microenvironment and facilitates neural repair.	Melentijevic et al. [141]

In summary, MSCs and their derivatives can achieve multi-dimensional protection against CIRI by regulating the “immune–mitochondrial” axis, and their preclinical therapeutic potential has been fully validated. To systematically present the progress in clinical translation of this therapy, Table 4 summarizes key ongoing or completed phase I/II clinical trials of MSC therapy for ischemic stroke, detailing the intervention protocols, preliminary efficacy, and safety outcomes of current clinical studies.

**Table 4:** Summary of clinical trials on MSCs and their exosomes for the treatment of ischemic stroke.

Trial name	Registration number	Trial stage	Cell origin	Results
TREASURE	NCT02961504	Phase II/III	Allogeneic BM-MSCs	High level of security
AMASCIS	NCT01678534	Phase II	Allogeneic AD-MSCs	High level of security
STARTING-2	NCT01716481	Phase III	Autologous BM-MSCs	High level of security
ISIS-HERMES	NCT00875654	Phase II	Autologous BM-MSCs	Improvement in motor function
Levy	NCT01297413	Phase I/II	Allogeneic MSCs	High level of security
SB623	NCT01287936	Phase I/IIa	Modified BM-MSCs	High level of security
MASTERS	NCT01436487	Phase II	MultiStem	High level of security

## Future perspectives

Although MSCs and their derivatives have demonstrated significant potential in treating CIRI, particularly by exerting neuroprotective effects through the dynamic modulation of the “immune-mitochondrial” axis, the complete scope of their biological functions and underlying mechanisms within the complex pathological milieu of CIRI remains to be fully elucidated. MSCs are known to possess multipotent differentiation capacity, potent paracrine functions, and immunomodulatory abilities. However, certain functions of MSCs in CIRI, such as the precise regulation of specific immune cell subsets, deeper impacts on mitochondrial quality control, and their overall contribution to neurovascular unit repair, have not been completely clarified. Future research should focus on systematically untangling the unrecognized functions of MSCs during different pathological stages of CIRI. For instance, it is crucial to investigate whether MSCs can intervene in emerging pathological processes, such as mitochondrial calcium homeostasis or the regulation of pyroptosis during the acute phase, potentially through the release of novel mitochondrial-derived peptides or specific non-coding RNAs. Such insights would provide a theoretical foundation for developing novel intervention strategies based on the functional properties of MSCs.

Furthermore, the clinical translation of MSC derivatives urgently requires the establishment of standardized methods for production and quality testing. The heterogeneity in preparation, storage, and characterization protocols leads to significant variations in the bioactivity and therapeutic efficacy of cell-free products such as exosomes, which can severely undermine their therapeutic effects and reproducibility. This represents a core bottleneck limiting their clinical application. Concurrently, unresolved controversies and issues of result heterogeneity persist in current MSC research. For instance, the limited in vivo survival capacity of intravenously administered MSCs in ischemic brain tissue may weaken long-term therapeutic effects. Additionally, due to differences in animal strains, modeling methods, and intervention timing, the therapeutic efficacy of MSCs exhibits marked heterogeneity across various CIRI animal models. These issues must be addressed in subsequent research to lay a solid foundation for clinical application.

The pathological progression of CIRI is characterized by distinct temporal and dynamic evolution. The specific roles of intrinsic molecular networks-including epigenetic regulation, metabolite fluctuations, and inter-organellar communication-within the injury cascade remain unclear. The therapeutic effects of MSCs might be achieved by acting on these newly identified dynamic nodes. For example, it warrants investigation whether MSCs or their exosomes can reshape the immune response by modulating the mitochondrial unfolded protein response, influencing the metabolic memory of immune cells, or altering the metabolomic profile of the local microenvironment. Identifying the key molecular events within these dynamic processes and defining the novel targets through which MSCs interact with them will be paramount. Intervention targeting these newly identified elements holds promise for overcoming the limitations of current therapeutic strategies, thereby advancing the development of next-generation pharmaceuticals and facilitating the transition from “cell therapy” to “precision medicine”.

CIRI represents a major global public health challenge due to its high disability and mortality rates. Its pathological mechanism is complex, involving a vicious cycle of multi-cellular, multi-pathway, and multi-temporal events, which results in scarce and limited effective clinical treatments. Within this challenging context, MSC therapy holds promise for making breakthrough contributions to the clinical management of CIRI, owing to its unique temporal characteristics and multi-target regulatory capabilities. Its potential lies not only in supplementing or serving as an alternative to existing thrombolytic and thrombectomy therapies to mitigate reperfusion injury per se, but more importantly, in offering long-term solutions for post-CIRI neurological deficits, chronic neuroinflammation, and impaired tissue healing. By systematically restoring mitochondrial function and remodeling the dysregulated immune network, MSC therapy may break the vicious cycle of “ischemia–reperfusion–subsequent injury,” thereby offering hope for protecting the ischemic penumbra and promoting neurovascular regeneration and functional recovery. In the future, through optimization of MSC administration timing, delivery routes, and formulation, combined with precision medicine approaches to identify optimal responder populations, therapeutic strategies utilizing MSCs and their derivatives are highly likely to become a pivotal force in combating the significant clinical challenge of CIRI, ultimately improving long-term neurological outcomes and the quality of life for stroke patients.

## Conclusion

CIRI is a complex pathological process involving the interplay of multiple pathways, including energy failure, oxidative stress, immune-inflammatory responses, and cell death, all of which follow a distinct temporal sequence. As a secondary injury specific to ischemic stroke, CIRI is characterized most distinctly by its acute onset and rapidly progressive damage, in contrast to neurological conditions such as neurodegenerative diseases. Unlike the slow, progressive damage seen in chronic disorders—marked by gradual mitochondrial dysfunction and long-term immune microenvironment imbalance—CIRI can trigger a cascade of events within a short timeframe. This cascade progresses from rapid energy depletion during the ischemic phase to mitochondrial collapse and oxidative stress burst in the acute reperfusion phase, and further to the full activation of an immune storm in the subacute phase. Within hours to days, this process leads to irreversible neuronal death in the ischemic penumbra. Coupled with the rapid disruption of the blood-brain barrier and swift infiltration of peripheral immune cells, it further accelerates the progression and expansion of brain damage. This unique pathological profile dictates that therapeutic strategies for CIRI cannot simply follow the intervention logic applied to chronic neurological disorders, nor can they be effectively blocked by single-target drugs. Instead, it necessitates establishing differentiated intervention principles tailored to its pathological progression. These principles include seizing the golden therapeutic window for rescuing the ischemic penumbra to achieve rapid efficacy, dynamically adjusting the therapeutic focus according to the core damaging features of different pathological stages, and simultaneously coordinating the regulation of intertwined pathological pathways such as metabolism, inflammation, and apoptosis. The dynamic, time-sequential regulatory role exerted by mesenchymal stem cells and their derivatives via the “immune-mitochondrial axis” aligns precisely with the therapeutic demands imposed by this unique pathological characteristic of CIRI. This alignment constitutes the core advantage distinguishing this approach from conventional treatments.

Mitochondrial dysfunction serves not only as a key driver in the acute phase of CIRI but also as a critical nexus linking metabolic disorders and immune imbalance. This review systematically elaborates that MSCs and their derivatives, as ideal therapeutic agents with dynamic regulatory capabilities, can mitigate CIRI through a “time-specific intervention” strategy. During the acute phase of reperfusion, MSCs effectively restore mitochondrial function in damaged neurons via multiple mechanisms, including direct mitochondrial transfer, regulation of mitochondrial dynamics, attenuation of oxidative stress, promotion of mitochondrial biogenesis, and improvement of energy metabolism. Consequently, cellular energy homeostasis is reestablished, and apoptotic signaling is suppressed.

More importantly, this improvement in mitochondrial function during the acute phase lays a solid foundation for reshaping the dysregulated immune network in the subacute and recovery phases. By ameliorating mitochondrial function, MSCs profoundly and indirectly remodel the post-CIRI immune network: promoting the polarization of microglia/macrophages towards an anti-inflammatory phenotype, inhibiting NLRP3 inflammasome activation, reducing neutrophil infiltration and NET formation, modulating pathogenic T-cell responses, and enhancing regulatory T-cell function. This sequential, multi-level, multi-target regulatory mechanism, mediated by the “mitochondria-immune” axis from the early to late stages, enables MSC-based therapy to break the vicious cycle between metabolic disturbance and immune dysregulation in CIRI, thereby achieving synergistic effects in neuroprotection and tissue healing.

Therefore, MSCs and their derivatives represent a novel therapeutic strategy that adopts an integrated “neuro-immune-metabolic” perspective and emphasizes the importance of timely intervention. This approach not only compensates for the limitations of traditional single-target drugs but also provides a new theoretical basis and translational direction for the clinical intervention of stroke and other neuroinflammatory diseases, underpinned by its “time-window-dependent” treatment concept.
